# Autophagy modulates tenogenic differentiation of cartilage-derived stem cells in response to mechanical tension via FGF signaling

**DOI:** 10.1093/stcltm/szae085

**Published:** 2024-11-29

**Authors:** Rui Zuo, Haoke Li, Chenhui Cai, Wen Xia, Jiabin Liu, Jie Li, Yuan Xu, Yi Zhang, Changqing Li, Yuzhang Wu, Chao Zhang

**Affiliations:** Department of Orthopedics, Xinqiao Hospital, Army Medical University, Chongqing 400037, People’s Republic of China; Department of Orthopedics, Xinqiao Hospital, Army Medical University, Chongqing 400037, People’s Republic of China; Department of Orthopedics, Xinqiao Hospital, Army Medical University, Chongqing 400037, People’s Republic of China; Department of Orthopedics, Xinqiao Hospital, Army Medical University, Chongqing 400037, People’s Republic of China; Department of Orthopedics, Xinqiao Hospital, Army Medical University, Chongqing 400037, People’s Republic of China; Department of Orthopedics, Xinqiao Hospital, Army Medical University, Chongqing 400037, People’s Republic of China; Department of Orthopedics, Xinqiao Hospital, Army Medical University, Chongqing 400037, People’s Republic of China; Chongqing International Institute for Immunology, Chongqing 401320, People’s Republic of China; Department of Orthopedics, Xinqiao Hospital, Army Medical University, Chongqing 400037, People’s Republic of China; Institute of Immunology, Army Medical University, Chongqing 400038, People’s Republic of China; Department of Orthopedics, Xinqiao Hospital, Army Medical University, Chongqing 400037, People’s Republic of China

**Keywords:** cartilage derived stem cells, autophagy, tendon repair, tenogenic differentiation, stem cell transplantation, FGF signaling, heterotopic ossification

## Abstract

**Background:**

In our previous study, we demonstrated that cartilage-derived stem cells (CDSCs) possess multi-differentiation potential, enabling direct bone-to-tendon structure regeneration after transplantation in a rat model. Therefore, the objective of this study is to investigate whether CDSCs are a suitable candidate for achieving biological regeneration of tendon injuries.

**Methods:**

Tenogenic differentiation was evaluated through cell morphology observation, PCR, and Western blot (WB) analysis. Autophagic flux, transmission electron microscopy, and WB analysis were employed to elucidate the role of autophagy during CDSC tenogenic differentiation. Cell survival and tenogenesis of transplanted CDSCs were assessed using fluorescence detection of gross and frozen section images. Heterotopic ossification and quality of tendon healing were evaluated by immunofluorescence, hematoxylin-eosin (H&E), and Safrinin O/Fast Green stains.

**Results:**

We found autophagy is activated in CDSCs when treated with cyclic tensile stress, which facilitates the preservation of their chondrogenic potential while impeding tenogenic differentiation. Inhibiting autophagy with chloroquine promoted tenogenic differentiation of CDSCs in response to cyclic tensile stress through activation of the Fgf2/Fgfr2 signaling pathway. This mechanism was further validated by 2 mouse transplantation models, revealed that autophagy inhibition could enhance the tendon regeneration efficacy of transplanted CDSCs at the patellar tendon resection site.

**Conclusion:**

Our findings provide insights into CDSC transplantation for achieving biological regeneration of tendon injuries, and demonstrate how modulation of autophagy in CDSCs can promote tenogenic differentiation in response to tensile stress both in vivo and in vitro.

## Graphical Abstract

**Graphical Abstract:**
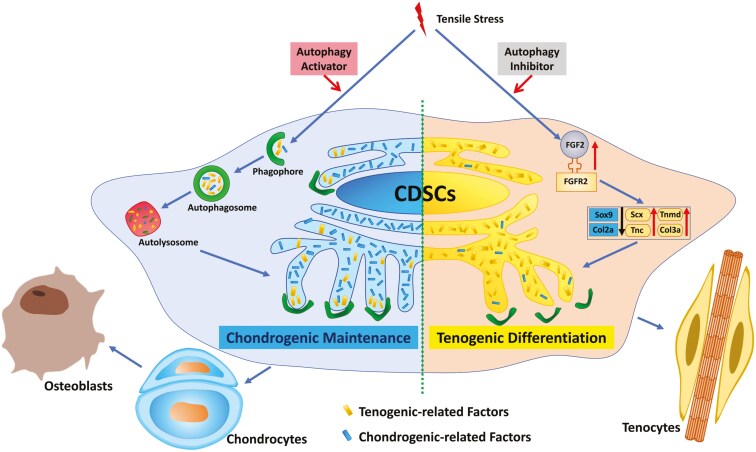

## Introduction

Tendons, the connective tissues responsible for transmitting muscle contractions to bones, possess remarkable structural strength.^1^ However, their intrinsic regenerative capacity is limited, necessitating surgical intervention upon tendon rupture.^2,3^ Unfortunately, post-operative tendon repair often results in scar tissue formation, increasing the risk of re-rupture.^4^ Achieving true non-scarring regeneration of ruptured tendons requires the targeted migration of a significant number of tenogenic stem cells to the injury site, followed by the reconstruction of collagenous fibrous structures.^5-7^ Despite this need, no established biological regenerative therapies currently exist for tendon rupture.^8^

Mesenchymal stem cells (MSCs) are a population of non-hematopoietic, self-renewing progenitor cells residing within the bone marrow. These cells demonstrate the ability to differentiate into various mesenchymal lineages, including bone, adipose tissue, cartilage, and stromal cells.^9^ While MSCs can be readily sourced from abundant tissues such as bone marrow, adipose tissue, peripheral blood, and umbilical cord,^10^ robust evidence supporting their long-term survival and differentiation into specific tissues following transplantation remains limited. Current understanding suggests that the primary function of transplanted MSCs lies in immunoregulation, promoting angiogenesis, and stimulating the migration and differentiation of endogenous stem cells rather than directly forming specific tissues themselves.^9,11,12^ It is now understood that tendon stem cells (TSCs), isolated from tendons in young individuals, exhibit typical stem cell characteristics but with a superior tenogenic differentiation capacity compared to MSCs. Additionally, TSCs demonstrate superior clonal formation and proliferation abilities.^13,14^ However, a significant drawback lies in the extremely limited source of TSCs, as they are absent in adult individuals. This limitation highlights the ongoing challenge of identifying suitable stem cells for effective biological regeneration of tendon injuries.

Interestingly, during embryogenesis, a bipotent progenitor co-expressing scleraxis (Scx) and Sox9 (Scx^+^/Sox9^+^) exhibits the potential to differentiate into both tendon and cartilage tissues.^15,16^ The fate of this progenitor is primarily dictated by the surrounding microenvironment, particularly mechanical forces. There is an increasing consensus suggesting that tensile forces promote tenogenic differentiation, while compressive forces favor chondrogenic and osteogenic differentiation pathways.^17-19^ Compared to tendons, cartilage offers a more abundant and purer source of tissue, with the capacity to maintain its chondrocyte phenotype for extended periods in adulthood, particularly in the case of costal cartilage.^20^ Cartilage-derived stem cells (CDSCs) can be readily isolated from adult costal cartilages.^21-23^ Given the shared lineage origin with Scx^+^/Sox9^+^ progenitors, it is highly conceivable that CDSCs represent their downstream progeny. However, no studies have hitherto investigated whether manipulating the mechanical stress and extracellular matrix microenvironment surrounding CDSCs can induce a shift in their inherent chondrogenic propensity towards tenogenic differentiation.

Our previous study demonstrated the successful application of costal CDSCs for regenerating the bone-tendon interface in a rat model.^24^ The transplanted CDSCs exhibited long-term viability and multilineage differentiation potential, generating cells of osteogenic, chondrogenic, and tenogenic lineages. These promising attributes position CDSCs as attractive candidates for future biological therapies targeting tendon injuries. However, several challenges require resolution before clinical translation can be achieved. A major concern lies in the potential for heterotopic ossification, the formation of bone tissue within the tendon area, following CDSC transplantation. This complication likely arises from the inherent chondrogenic potential of CDSCs. Therefore, future studies should investigate strategies to mitigate chondrogenesis while simultaneously promoting the tenogenic differentiation of CDSCs.

Autophagy is a “self-protecting” cellular process to degrade and recycle damaged and aged proteins and organelles, thereby preventing cell damage. This process contributes to the homeostasis of adult stem cells in terms of their quiescence, self-renewal, and differentiation.^25-27^ In our previous study, we demonstrated that autophagy activation maintains the chondrogenic potential of cartilage stem cells and inhibits inflammation-mediated endplate degradation.^28^ However, the involvement of autophagy in tenogenic differentiation of CDSCs has not been previously reported.

The present study investigated the potential of enhancing tenogenic differentiation of CDSCs under tensile stress conditions by modulating autophagy. We employed a series of in vitro and in vivo experiments to demonstrate that autophagy inhibition in CDSCs subjected to tensile stress promotes their tenogenic differentiation through the activation of FGF signaling. Taken together, these findings provide valuable insights for the development of a biological regeneration approach utilizing stem cell transplantation for tendon injuries.

## Methods

### Ethical approval

All animal experiments were conducted in accordance with the guidelines set by the Experimental Animal Welfare and Ethics Committee of Army Medical University (AMUWEC20234900). Animals were maintained in a rigorously controlled environment, ensuring consistent temperature, natural ventilation, and a regular light-dark cycle to mimic their natural circadian rhythm. Specialized food and water were provided ad libitum to meet all their nutritional needs.

### Isolation and culture of mouse CDSCs

Neonatal B6-G/R mice (Strain No. T006163) were obtained from GemPharmatech (Nanjing, China), while neonatal wild-type mice were procured from the Laboratory Animal Center of Army Medical University. One-week-old B6-G/R or wild-type mice were euthanized by cervical dislocation under sterile conditions. Subsequently, costal cartilages were dissected from the euthanized mice. Careful dissection procedures ensured the removal of ribs, sternum, and soft tissues to isolate individual costal cartilages. The isolated cartilages were disinfected by immersion in a 0.5 g/L iodophor solution for 30 seconds, followed by 3 washes with 1 × phosphate-buffered saline (PBS). The digestion process involved shaking the cartilages at 37°C and 80 rpm in a constant temperature shaker for 40 minutes in a solution of 0.2% type II collagenase (enzyme volume 20 times that of the tissue volume). The supernatant was then discarded. The remaining cartilage fragments were washed 3 times with 1 × PBS and subjected to a second digestion step with 0.2% type II collagenase under the same shaking conditions for 60 minutes. The resulting supernatant containing the isolated cells was collected, and the remaining cartilage tissues were washed repeatedly with 1 × PBS to obtain single-cell suspensions. These cell suspensions were centrifuged at 4°C and 400×*g* for 5 minutes. The supernatant was removed, and the cells were resuspended in a growth medium consisting of Dulbecco’s Modified Eagle’s Medium/Ham’s F12 Nutrient Mixture (DMEM/F12) supplemented with 10% fetal bovine serum (FBS) and 1× penicillin-streptomycin solution. Finally, the cells were seeded at a density of 2× 10^4^ cells/cm^2^ in a 25 cm^2^ culture plate and incubated at 37°C with 5% CO_2_. After 24 hours, non-adherent cells were removed, and the remaining adherent cells were designated as passage 0 (P0). Upon reaching 100% confluence, the cells were sub-cultured, and their passage number was increased to P1.

### Flow cytometry for cell characterization

The characteristics of CDSCs were validated using the Mesenchymal Stem Cell (Mouse) Surface Marker Identification Kit (MUXMX-09011, Cyagen). This kit allows for the identification of positive markers (CD90.2, CD44, and CD29) and negative markers (CD34 and CD31), with Rat IgG2b, κ serving as the isotype control. Flow cytometry analysis was conducted following the manufacturer’s instructions. P3 CDSCs were harvested and resuspended at a density of 3 × 10^6^ cells/mL. Subsequently, 2 μL of each primary antibody was added to separate aliquots of 100 μL cell suspension. The cells were then incubated at 4 °C for 30 minutes, followed by 2 washes with 200 μL buffer each. After centrifugation at 4 °C and 250×*g* for 5 minutes, the supernatant was discarded. Next, 100 μL of buffer containing 2 μL of FITC-Goat anti-rat IgG antibody was added to each tube. The cells were resuspended and incubated at 4 °C for 30 minutes. After 2 washes with 200 μL buffer each, the cells were resuspended in 300 μL of buffer. Flow cytometry analysis was performed using the Gallios flow cytometer (Beckman Coulter) with FlowJo software (version 10.0.7, BD company) for data analysis.

### In vitro tension experiments

P3 CDSCs were resuspended in DMEM/F12 medium supplemented with 10% FBS and seeded at a density of 1 × 10^5^ cells per well in 6-well BioFlex culture plates (BF-3001, Flexcell). The plates were incubated at 37 °C with 5% CO_2_ and 1% O_2_ for 12 hours. Subsequently, the culture medium was removed, and each well was supplemented with 2 mL of fresh medium containing either 2 μmol/L rapamycin (R8140, Solarbio), 40 μmol/L chloroquine (C9720, Solarbio), or 100 nM/L AZD4547 (SF5440, Beyotime). The plates were then transferred to the Flexcell 3D Cell Culture System (FX-5000T) and subjected to 5% tensile stress at 1 Hz frequency. Cell morphology was monitored and captured at different time points.

### Western-blot

P3 mouse CDSCs were subjected to 72 hours of tensile stress treatment, either alone or in combination with rapamycin, chloroquine, or AZD4547. Subsequently, the cells were harvested, and proteins were extracted on ice using Western and IP Lysis Buffer (P0013, Beyotime). WB analysis was performed following a previously established standard protocol. Briefly, the protein concentration was quantified using a BCA Protein Assay Kit (P0010, Beyotime). The total protein lysates were then separated on an SDS-PAGE gel (P0012AC, Beyotime) and transferred to 0.22 μm PVDF membranes (ISEQ00010, Millipore). The membranes were blocked with QuickBlock Blocking Buffer (P0231, Beyotime) for 15 minutes at room temperature, followed by overnight incubation with primary antibodies at 4°C.

WB analysis was performed using primary antibodies specific for the following proteins: collagen type III (Col III; 1:800 dilution; catalog no. AF6531; Beyotime), scleraxis (Scxa; 1:500 dilution; catalog no. ab58655; Abcam), tenomodulin (Tnmd; 1:500 dilution; catalog no. ab203676; Abcam), Sox9 (1:5000 dilution; catalog no. ab185230; Abcam), p62 (SQSTM1; 1:1000 dilution; catalog no. ab56416; Abcam), LC3 (1:1000 dilution; catalog no. ab48394; Abcam), fibroblast growth factor 2 (Fgf2; 1:1000 dilution; catalog no. DF6038; Affinity), fibroblast growth factor receptor 2 (Fgfr2; 1:1000 dilution; catalog no. AF0159; Affinity), and β-actin (housekeeping control; 1:1000 dilution; catalog no. AF5001; Beyotime). Secondary horseradish peroxidase (HRP)-conjugated antibodies against mouse (1:1000 dilution; catalog no. A0216; Beyotime) and rabbit (1:1000 dilution; catalog no. A0208; Beyotime) IgG were used. Protein expression levels were normalized to β-actin.

### RT-PCR analysis

P3 passage mouse CDSCs were treated with tensile stress for 72 hours, with and without the addition of rapamycin, chloroquine, and AZD4547. Following the tensile stress treatment, the cells were used for RT-PCR analysis. Total RNA was extracted from the cells using an RNAiso Plus Kit (9108, Takara). Five micro grams of the total RNA was then reverse transcribed into cDNA using PrimeScript RT reagent Kig with gDNA Eraser (RR047A, TaKaRa). The PCR amplification protocol consisted of an initial denaturation step at 95°C for 30 seconds, followed by 40 cycles of amplification. Each cycle of amplification included denaturation at 95°C for 5 seconds and annealing/extension at 60°C for 30 seconds. TB Green Premix Ex Taq II (2x) mix from TaKaRa (RR820A, Takara) was used in a 20 μL reaction volume for the PCR. The PCR primer sequences of mouse were as follows:

SRY-box transcription factor 9 (Sox9):

Forward: AGGAAGTCGGTGAAGAACGG

Reverse: GGACCCTGAGATTGCCCAGA

Collagen type Ⅲ alpha 1 (Col3a1)

Forward: TGACTGTCCCACGTAAGCAC

Reverse: GAGGGCCATAGCTGAACTGA

Tenomodulin (Tnmd):

Forward: CACTTCTGGCCCGAGGTATC

Reverse: AGTAGATGCCAGTGTATCCATTTTT

Collagen type Ⅱ alpha 1 (Col2a1):

Forward: GCCAGGATGCCCGAAAATTAG

Reverse: CGCACCCTTTTCTCCCTTGT

Scleraxis (Scx):

Forward: GCCACTGAAGAGTCACGGAG

Reverse: TAGAGTCAAGCCATCACCCG

Tenascin C (Tnc):

Forward: CAGTTTCCTGGACGGCATCG

Reverse: CCTCAAGGGCTTTGTTTGGTG

Fibroblast growth factor receptor 2 (Fgfr2):

Forward: CCTGCGGAGACAGGTTTCG

Reverse: TTGCCCAGCGTCAGCTTATC

Fibroblast growth factor receptor 1 (Fgfr1):

Forward: GACTCTGGCCTCTACGCTTG

Reverse: GTAGGGAGCTACAGGGTTTGG

Bone morphogenetic protein 2 (Bmp2):

Forward: TTGGAACTCCAGACTGTGCG

Reverse: GAGGGTAAGACTGTGCTCCG

Bone morphogenetic protein 4 (Bmp4):

Forward: TAAACCGTCTTGGAGCCTGC

Reverse: TGGTGTCTCATTGGTTCCTGC

Fibroblast growth factor 2 (Fgf2):

Forward: GGCTGCTGGCTTCTAAGTGT

Reverse: TCTGTCCAGGTCCCGTTTTG

Fibroblast growth factor 4 (Fgf4):

Forward: GGTGAGCATCTTCGGAGTGG

Reverse: GTCCGCCCGTTCTTACTGAG

Transforming growth factor beta 2 (Tgfb2):

Forward: GGCTCATTGGGCAGCTTTTG

Reverse: AAGCGGAAGACCCTGAACTC

Transforming growth factor beta 3 (Tgfb3):

Forward: TTTGCGGAGGACGGAGTAAC

Reverse: ACAGTCACCAGCATCTCAGC

SMAD family member 3 (Smad3):

Forward: AACCCAAACTTTCTACTGCCAC

Reverse: CGCCCGAACTTCGCTTTT

SMAD family member 2 (Smad2):

Forward: TTGCTGTTGTTGTTGTTTAAGGA

Reverse: AGATGCCAGTAAGATTCGGTTGA

Growth differentiation factor 5 (Gdf5):

Forward: AAGAACCTCAAGGCTCGCTG

Reverse: AGGGTCTGAATGACTGCGTG

Growth differentiation factor 6 (Gdf6):

Forward: TTACCAGCCAGCAATAGCCC

Reverse: CTGTACCCCAGTTTCCACCC

Growth differentiation factor 7 (Gdf7):

Forward: CATGATGTCGCTTTACAGGAGC

Reverse: CCGCCGTTTCGTCTTGA

### Autophagic flux analysis by LV-mRFP-GFP-LC3 transduction

CDSCs were resuspended and seeded in 6-well plates at a density to achieve 30%-40% confluence. Cells were then infected with LV-mRFP-GFP-LC3 at a multiplicity of infection (MOI) of 10 (HB-LP2100001, Hanbio) following the manufacturer’s instructions. The culture medium was replaced with fresh medium 24 hours post-infection. After an additional 72 hours of culture, the medium was again replaced with fresh medium containing 6 μg/mL puromycin for selection for an additional 48 hours. Green and red fluorescent puncta represented phagophores and autolysosomes, respectively. Merged yellow puncta indicated autophagosomes.

### Transmission electronic microscope

Following in vitro treatments, CDSCs from each group were resuspended, with 3 × 10^5^ cells harvested for analysis. After fixation in 3% glutaraldehyde and dehydration in ethanol solutions, the samples were observed by transmission electron microscopy (TEM; JEM-1400PLUS, Olympus, Japan) at 80 kV.

### Type I collagen extraction

Type I collagen was extracted from the tail tendons of 12-week-old Sprague-Dawley rats (Tengxin Biotechnology) using the previously described acid extraction method.^29^ Briefly, the rats were euthanized by cervical dislocation under an approved protocol, and the tail tendons were aseptically dissected. The tendons were then sheared into 0.5 mm × 0.5 mm pieces and incubated in a 50-fold volume of 0.5% (v/v) acetic acid solution at 4°C for 72 hours. Following centrifugation at 8500 *g* for 15 minutes, the supernatant containing the extracted collagen was collected and salted-out with a 4-fold volume of 12.5% NaCl solution for 2 hours. Finally, the collagen-rich sediment was collected after another centrifugation at 8500 *g* for 15 minutes and dissolved in a 10-fold volume of 0.1 M HCl solution.

### Fabrication of decellular tendon scaffold and cell-gel

Decellularized tendon scaffolds (DTS) were obtained from the tail tendons of 12-week-old rats using a previously described decellularization protocol.^24^ The pH of the isolated type I collagen solution was then adjusted to 7.0 on ice using 1 M NaOH. P3 CDSCs were resuspended in this collagen solution at a density of 3 × 10^6^ cells/mL. Pre-straightened DTS were inserted into 3 mm diameter transparent plastic tubes. The cell-collagen mixture was then injected into the tubes to encapsulate the DTS. The constructs were incubated at 37 °C for 2 hours to allow for collagen polymerization. Subsequently, the cell-laden DTS were removed from the tubes, and excess liquid was removed by gentle squeezing. Finally, the cell-encapsulated DTS were cut into 1 cm segments for use in mouse tendon regeneration models.

### Mouse patellar tendon resection and regeneration model

Eight-week-old male mice (Tengxin Biotechnology) were anesthetized using intraperitoneal injection of 500 mg/kg tribromoethanol. Following anesthesia, the right knee of each mouse was shaved and a 1 cm incision was made to expose the patella, patellar tendon, and tibial tubercle. A standardized surgical approach was employed to resect the entire patellar tendon, the lower third of the patella (approximately 0.5 mm), and a portion of the tibial tubercle (approximately 0.5 mm). Subsequently, a 1 cm long cell-encapsulated DTS was transplanted to the surgical defect and secured to the patella and tibia using 6-0 nylon sutures. The incision was then closed using additional 6-0 nylon sutures. For designated experimental groups, mice received daily intraperitoneal injections of chloroquine (60 mg/kg) or rapamycin (4 mg/kg), or daily intragastric gavage of AZD4547 (12.5 mg/kg) from post-operative days 3-17. The concentration of intraperitoneal injections of chloroquine and rapamycin was reported in our previous published study,^28^ while concentration of AZD4547 was according to manufacture’s recommended that effectively inhibited FGFR family.

### Tissue processing and histological staining

Mice in each group were euthanized by cervical dislocation at designated time points. The right knee joints were then dissected and photographed with or without a 488 nm laser illumination (3415RG, LUYOR) for fluorescence imaging. Subsequently, the knee joints were fixed in 4% formalin for 24 hours, followed by decalcification in 10% EDTA solution at 37 °C and 80 rpm in a constant temperature shaker for 48 hours. The decalcified joints were then sagittally sectioned, and images of the sagittal surfaces were captured with the 488 nm laser. For histological analysis, knee joint tissues were embedded in OCT compound (4583, Sakura) and sectioned at a thickness of 8 μm. The sections were then stored frozen at −20°C. Staining with DAPI (P0131, Beyotime), hematoxylin and eosin (H&E, G1120, Solarbio), and Safranin O/Fast Green (G1371, Solarbio) was performed according to the manufacturers’ instructions.

### Immunofluorescence

Frozen sections of mouse knee joints from each group were first rewarmed to room temperature. Antigen retrieval was then performed by incubating the sections in Antigen Retrieval Solution (C1035, Solarbio) for 5 minutes. Following permeabilization with 1% Triton X-100 for 2 minutes, the sections were washed 3 times with PBS. Subsequently, they were incubated with primary antibodies diluted in goat serum at 4 °C overnight. The primary antibodies used targeted Tenascin-C (Tnc, 1:150 dilution, ab108930, Abcam), Collagen X (Col X, 1:200 dilution, ab182563, Abcam), and Osterix (Osx, 1:200 dilution, ab209484, Abcam). After washing the sections three times with PBS, they were incubated with Alexa Fluor 594-conjugated goat anti-rabbit secondary antibodies for 1 hour at room temperature in the dark. Finally, the sections were stained with DAPI for nuclear counterstaining. All fluorescence images were captured using a fluorescent microscope (IX73, Olympus).

### Histological assessments

Each section was evaluated using the Astrom and Movin semi-quantitative grading scale, incorporating modifications proposed by Maffulli.^30^ This scale assessed the following parameters: fiber structure, fiber arrangement, nuclei roundness, cell density, and the presence of heterotopic ossification. A scoring system was employed, where 0 indicated normal morphology, 1 indicated slight abnormality, 2 indicated moderate abnormality, and 3 indicated marked abnormality.

### Statistical analyses

All data were presented as mean ± standard deviation (SD) from at least 3 independent experiments. Fluorescence intensity was quantified using ImageJ software. For comparisons between 2 groups, a 2-tailed, unpaired Student’s *t*-test was employed. GraphPad Prism v.6.0a was used for all statistical analyses. A *P*-value < .05 was considered statistically significant (*P < .05, **P < .01, ***P < .001, ****P < .0001). All data points represent biological replicates unless otherwise specified in the figure legends. Statistic analyzed data have showed in charts of each figure, and uploaded in the supplementary material.

## Results

### Autophagy influences tenogenic differentiation of tension-treated CDSCs

**Figure 1.**
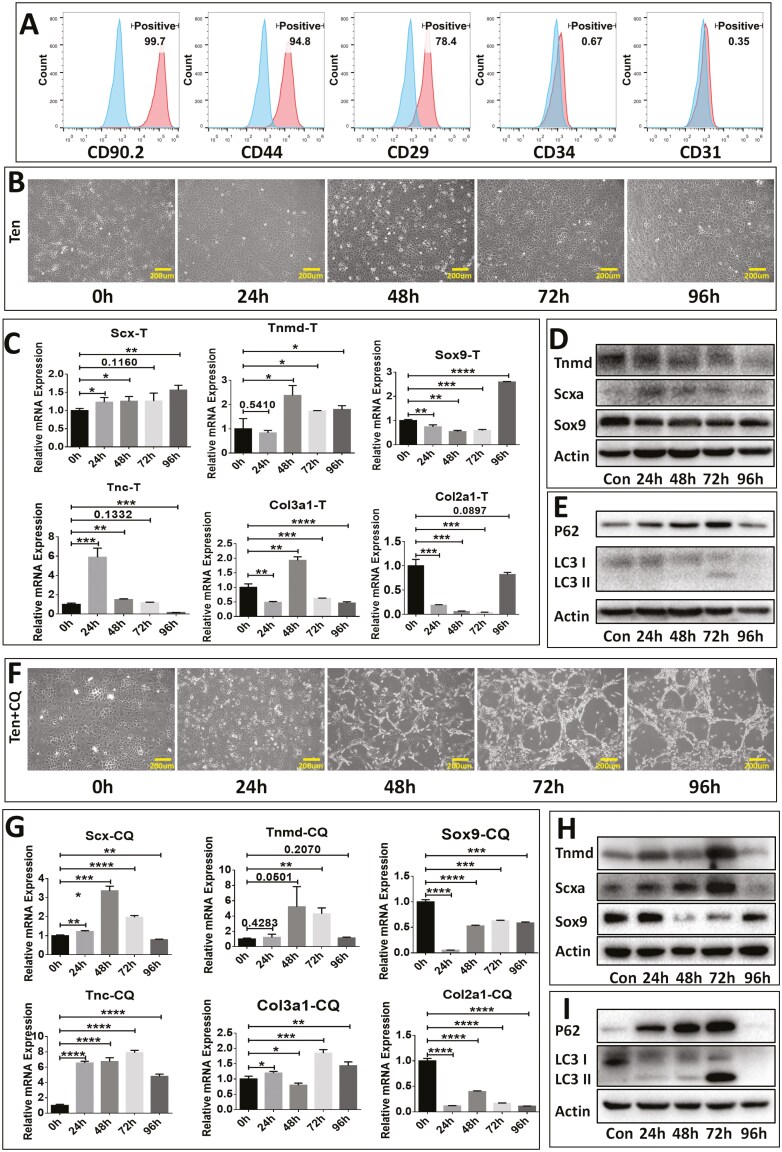
Autophagy influences the tenogenic differentiation of cyclic tensile stress-treated CDSCs. (A) Flow cytometry analysis was performed to evaluate the expression of mouse mesenchymal stem cell-related markers CD90.2, CD44, CD29, and CD34 in P3 passage mouse CDSCs. (B) Time-lapse images show the morphological changes of CDSCs under cyclic tensile stress (5%, 1HZ). (C-D) PCR (C) and Western Blot (D) analyses were conducted to assess the expression levels of tenogenic and chondrogenic-related markers in CDSCs treated with cyclic tensile stress at different time points (*n* = 3). T represents cyclic tension stress. (E) Western blot analysis was performed to examine autophagy-related marker expression in CDSCs treated with cyclic tensile stress at different time points. (F) Time-lapse images demonstrate the cellular morphology changes of CDSCs under both cyclic tensile stress and chloroquine treatment (40 μmol/L). (G-H) PCR (G) and Western Blot (H) analyses were carried out to determine the expression levels of tenogenic and chondrogenic-related markers in CDSCs subjected to both cyclic tensile stress and chloroquine treatment at different time points(*n* = 3). CQ denotes chloroquine. (I) Western blot analysis was conducted to investigate autophagy-related marker expression in CDSCs treated with cyclic tensile stress and chloroquine at different time points. Data are mean ± SD. *, *P* < .05; **, *P* < .01; ***, *P* < .001; ****, *P* < .0001.

To characterize the surface marker profile of mouse CDSCs, flow cytometry analysis was performed. The results demonstrated that over 75% of the CDSCs expressed established mesenchymal stem cell markers, including CD90.2, CD29, and CD44. Conversely, less than 1% of the cells expressed the negative markers CD31 and CD34 (Figure 1A).

Since tendons primarily transmit muscle contraction to bone, we sought to explore whether tensile stress could promote tenogenic differentiation in CDSCs. Following stimulation by cyclic tensile stress, CDSCs exhibited a gradual morphological change from a cobblestone-like appearance with irregular alignment to an elongated and aligned morphology oriented along the direction of stress (Figure 1B). However, this altered morphology did not match the characteristic shape of tendon stem cells. To further explore this phenomenon, we examined the expression of tenogenic markers through PCR and Western blot analysis. Regarding the expression results of Scx, Tnmd, Tnc, and Col3a1 markers, statistically remarkable and consistently increase (relative expression level >2 and over 48 hours) in the levels of these markers was not been observed. Interestingly, exposure to tensile stress resulted in a transient decrease in the mRNA levels of chondrogenic markers Sox9 and Col2a1 within 72 hours, followed by a return to high expression levels after 96 hours. Western blot analysis revealed minimal changes in the protein level of Sox9 upon exposure to tensile stress (Figure 1C and D). The discrepancy between mRNA and protein expression profiles of Sox9 within 96 hours suggests that CDSCs adapt to tensile stress by a transiently decreasing Sox9 transcription. Once CDSCs fully acclimate to tensile stress, Sox9 transcription is allowed to recover. As tensile stress does not alter the differentiation potential of CDSCs, the protein levels of Sox9 are maintained. These findings suggest that in vitro tensile stress alone exhibits limited ability to induce tenogenic differentiation of CDSCs. This may indicate the activation of a homeostasis maintenance mechanism in response to the stress stimulus.

Building upon our previous finding that autophagy activation supports the chondrogenic phenotype in cartilage endplate stem cells, we explored its potential role in maintaining CDSC homeostasis under cyclic tensile stress. Western blot analysis revealed persistent autophagy activation in tension-treated CDSCs, as indicated by decreased levels of autophagy markers P62 and LC3 II at various time points (Figure 1E). To investigate autophagy’s role further, chloroquine, an inhibitor that blocks autophagosome-lysosome fusion, was added to the medium of tension-treated CDSCs. This resulted in significant morphological changes within 48 hours. Originally cobblestone-like CDSCs transformed into spindle-shaped cells resembling previously reported tendon stem cells (Figure 1F). Furthermore, PCR and Western blot analyses demonstrated a significant upregulation of tenogenic markers and a downregulation of chondrogenic markers within 72 hours. However, these changes were likely transient due to potential chloroquine degradation or insufficient concentration. We observed a decrease in tenogenic markers while chondrogenic markers recovered to near original levels after 96 hours (Figure 1G and H). Consistent with this, Western blot analysis confirmed autophagy reactivation at 96 hours, evidenced by the reappearance of P62 and LC3 proteins (Figure 1I).

### Rapamycin/chloroquine modulated the autophagic status of tension-treated CDSCs, thereby influencing their tenogenic differentiation

**Figure 2.**
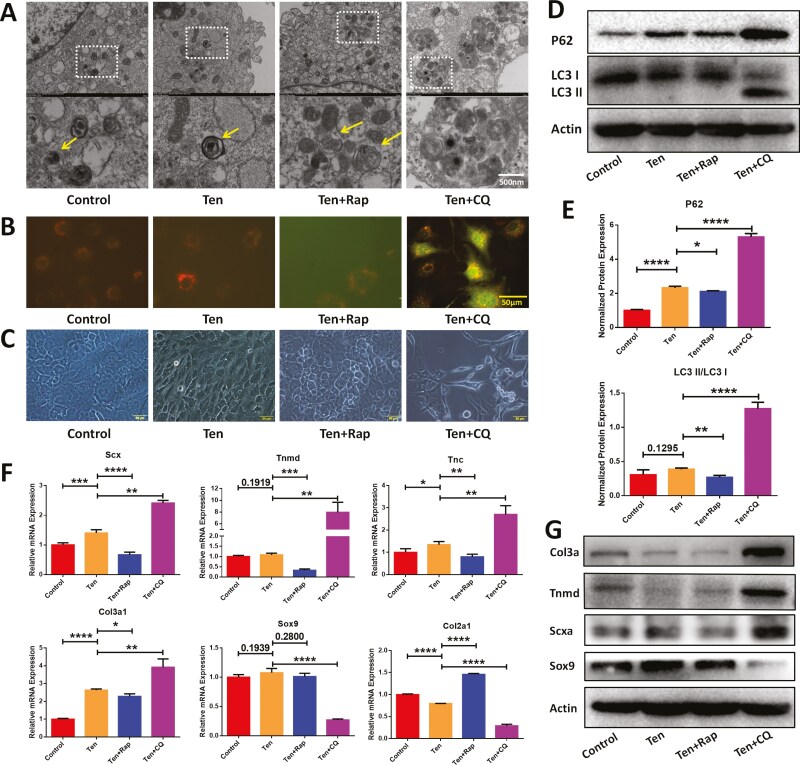
Rapamycin/chloroquine treatment altered the autophagy status of tension-treated CDSCs, thereby influencing tengogenic differentiation. (A) TEM images showing autophagosomes in each group: control (normal cultured CDSCs), Ten (cyclic tensile stress treated CDSCs), Ten + Rap (cyclic tensile stress and rapamycin-treated CDSCs), Ten + CQ (cyclic tensile stress and chloroquine treated CDSCs). Scale bar = 500 nm. (B) Fluorescent images depicting autophagy flux in each group. Scale bar = 50 μm. Merged GFP and RFP dots indicate autophagosomes. (C) Cell morphology of CDSCs observed in each group. Scale bar = 50 μm. (D-E) Western blot analysis was performed to assess the expression levels of autophagy-related markers in each group (*n* = 3). (F-G) PCR (F) and Western blot (G) analyses were conducted to evaluate the expression levels of tenogenic and chondrogenic-related markers in CDSCs from each group (*n* = 3). Data are mean ± S.D. *, *P* < .05; **, *P* < .01; ***, *P* < .001; ****, *P* < .0001.

To compare the effects of autophagy modulation on tenogenic differentiation of tension-treated CDSCs, we supplemented the culture medium with either rapamycin (Ten + Rap) or chloroquine (Ten + CQ) and analyzed the cells at 72 hours. Rapamycin is an mTOR inhibitor with specificity, which induces autophagy. The Autophagy status of CDSCs in each group was assessed using TEM and an autophagy flux lentivirus system. Both TEM and autophagy flux analysis revealed minimal autophagosome formation in the Control, Ten, and Ten + Rap groups. Conversely, the Ten + CQ group displayed a significant increase in autophagosome number, evidenced by the presence of large autophagosomes in TEM images and the appearance of yellow fluorescent puncta representing autophagic flux (Figure 2A and B). Additionally, morphological examination revealed that CDSCs in the Ten + CQ group adopted a spindle-shaped morphology resembling reported tendon stem cells, while cells in the Ten + Rap group reverted to a cobblestone-like appearance with increased cell density (Figure 2C). Western blot analysis of P62 and LC3 expression further confirmed the effectiveness of chloroquine in inhibiting autophagy (Figure 2D and E).

We further investigated the impact of autophagy modulation on the expression of tenogenic and chondrogenic markers. PCR and Western blot analyses revealed that rapamycin-induced autophagy facilitated the maintenance of chondrogenic characteristics in CDSCs. This was evidenced by decreased expression of tenogenic markers (Scx, Tnmd, Tnc, and Col3a1) and maintained expression of chondrogenic markers (Col2a1 and Sox9) (Figure 2F and G). Conversely, chloroquine, which inhibited autophagy, promoted the expression of tenogenic markers (Scx, Tnmd, Tnc, Col3a1) while reducing the expression of chondrogenic markers (Col2a1 and Sox9).

In summary, our findings demonstrated that rapamycin-induced autophagy could support the maintenance of both the characteristic morphology and the chondrogenic phenotype of CDSCs under tensile stress. Conversely, chloroquine treatment could inhibit autophagy and promote the transformation of CDSCs into cells with a morphology resembling tendon stem cells, accompanied by a concomitant upregulation of tenogenic markers.

### Chloroquine promote the tenogenic differentiation of tension-treated CDSCs by activating FGF signaling

**Figure 3.**
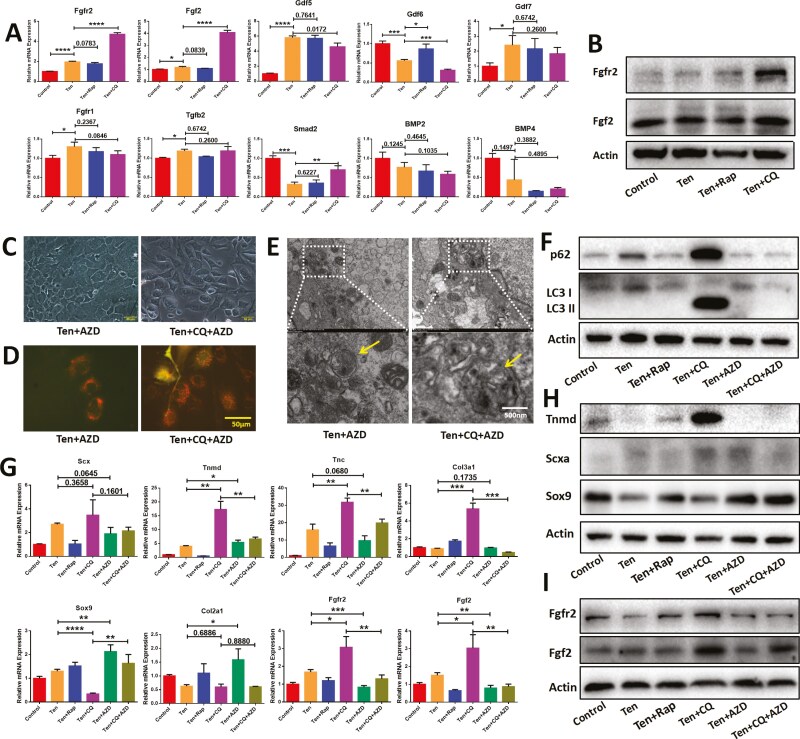
Autophagy inhibition promotes tenogenic differentiation of tension-treated CDSCs through activation of FGF signaling. (A) PCR analysis of tenogenic differentiation-related signaling pathways of CDSCs in each group (*n* = 3). (B) Western Blot analysis for FGF signaling. (C) Cell morphology of CDSCs in Ten + AZD and Ten + CQ + AZD groups. Ten + AZD, cyclic tensile tress and AZD4547 (100 nM/L) treated CDSCs; Ten + CQ + AZD, cyclic tensile tress, chloroquine and AZD4547 treated CDSCs. (D) Fluorescent images of autophagy flux in Ten + AZD and Ten + CQ + AZD groups. Scale bar = 50 μm. (E) TEM images of autophagosomes in Ten + AZD and Ten + CQ + AZD groups. Scale bar = 50 μm. Merged GFP and RFP dots indicate autophagosomes. (F) Western Blot analysis for the autophagy-related markers in each group. (G-I) PCR (G) and Western Blot (H,I) analyses for the tenogenic, chondrogenic-related markers and Fgfr2, Fgf2 (*n* = 3). Data are mean ± S.D. *, *P* < .05; **, *P* < .01; ***, *P* < .001; ****, *P* < .0001.

To elucidate the mechanisms underlying autophagy-regulated tenogenic differentiation of tension-treated CDSCs, we investigated gene expression profiles associated with tenogenic differentiation and tendon development signaling pathways. Genes with undetectable expression were excluded from further analysis. The remaining genes and their expression patterns are presented in Figure 3A. Notably, the PCR results suggested a potential role for fibroblast growth factor (FGF) signaling in the pro-tenogenic differentiation effects observed upon autophagy inhibition in tension-treated CDSCs. This hypothesis was further supported by Western blot analysis, which revealed a significant increase in the protein levels of Fgf2 and Fgfr2 in the Ten + CQ group (Figure 3B).

To further investigate the role of FGF signaling in autophagy-mediated tenogenic differentiation, we employed AZD4547 (AZD), a potent inhibitor of the FGFR family. Notably, treatment with AZD (both Ten + AZD and Ten + CQ + AZD groups) resulted in a morphological recovery of the CDSCs, with cells reverting to a cobblestone-like appearance (Figure 3C). These findings suggested that AZD4547 counteracted the pro-tenogenic effects and autophagy inhibition induced by chloroquine. Autophagy flux analysis, TEM, and Western blotting confirmed the reactivation of autophagy in CDSCs treated with Ten + CQ + AZD compared to those treated with Ten + CQ alone (Figure 3D-F). Furthermore, inhibition of FGF signaling by AZD4547 in the Ten + CQ + AZD group led to decreased expression of tenogenic markers and a concomitant increase in chondrogenic markers compared to the Ten + CQ group (Figure 3G and H). Collectively, our data demonstrated that chloroquine promotes tenogenic differentiation of tension-treated CDSCs by suppressing autophagy and activating FGF signaling. Importantly, inhibition of FGF signaling abrogated these effects.

### Effects of post-transplant autophagy or FGF signaling interventions on tendon regeneration following transplantation of untreated CDSCs

**Figure 4.**
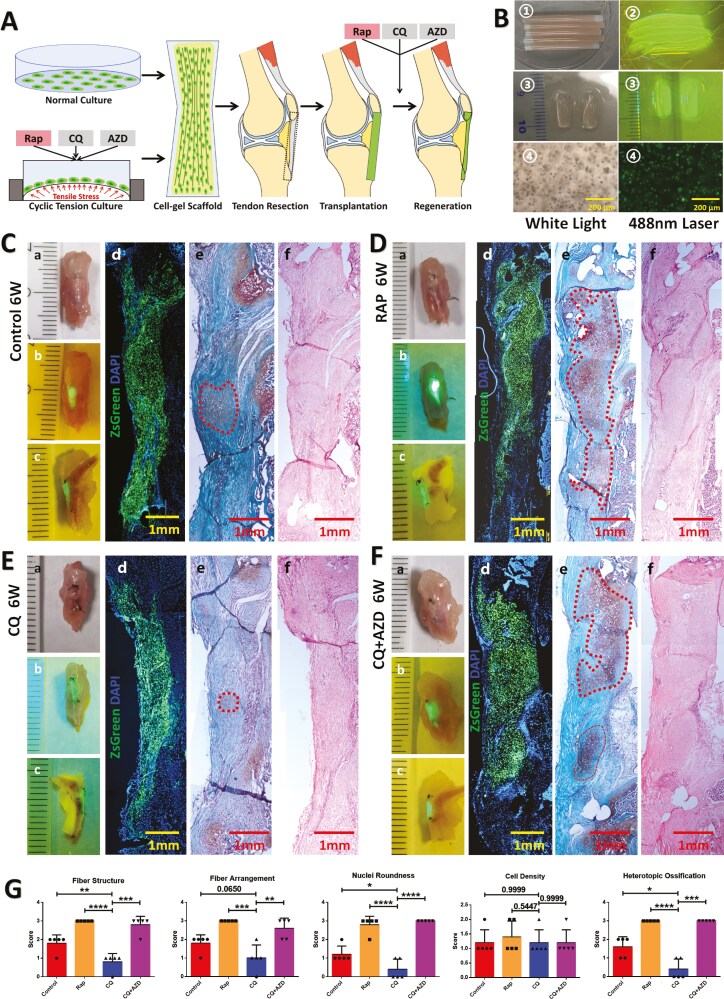
Effects of post-transplant autophagy or FGF signaling interventions on tendon regeneration of untreated CDSCs. (A) Flowchart illustrating the 2 transplantation models. Model 1, untreated CDSCs presentative gross and microscopic images of different groups that received untreated CDSCs transplantation after 6 weeks are shown. Gross images of the knee joint were captured under white light (a), 488 nm laser illumination (b, c). Microscopic images depict merged fluorescent staining (d), Safranin O staining (e), and H&E staining (f) of frozen sections. The scale bar represents 1 mm. Control group (C), transplant untreated CDSCs, without post-transplant interventions; Rap group (D), transplant untreated CDSCs, with post-transplant rapamycin injection; CQ group (E), transplant untreated CDSCs, with post-transplant chloroquine injection; CQ + AZD group (F), transplant untreated CDSCs, with post-transplant chloroquine and AZD4547 injection. (G) Histological scores of 5 parameters to evaluate tendon regeneration of stained sections (*n* = 5). Data are mean ± S.D. *, *P* < .05; **, *P* < .01; ***, *P* < .001; ****, *P* < .0001. Images of C-F are representative of 5 independent experiments.

To investigate the in vivo impact of autophagy on CDSC tenogenesis and explore whether inhibiting autophagy could promote tenogenesis of transplanted CDSCs at the tendon resection site through FGF signaling activation, we designed 2 transplantation models (Figure 4A). In Model 1, untreated CDSCs cultured in vitro were transplanted into the patellar tendon resection site, with the hypothesis that the local tension microenvironment would stimulate the post-transplantation CDSCs. Subsequently, rapamycin, chloroquine, and AZD4547 were administered intraperitoneally or intragastrically. In Model 2, pretreated CDSCs from each group were transplanted followed by post-transplantation injection of the same drugs (rapamycin, chloroquine, and AZD4547) (Figure 4A). The procedures for fabricating cell-gel wrapped DTS are illustrated in Figure 4B. Notably, the cells embedded within the collagen gel exhibit excellent viability.

Evaluation of transplanted cells across all groups revealed robust survival at 6 weeks post-transplantation. This was evidenced by continuous green fluorescence observed from the patella to the tibial tubercle in both macroscopic and microscopic images upon exposure to 488 nm laser illumination (Figure 4C and F). However, microscopic examination revealed diverse outcomes in terms of tendon regeneration across the groups. The control group (no post-transplantation interventions) exhibited a substantial area of heterotopic ossification at the center of the transplant site, demarcated by the red dotted line and confirmed by Safranin O staining (Figure 4C). Notably, the Rapamycin group (administered intraperitoneal rapamycin injections) displayed a significantly enlarged area of heterotopic ossification compared to the Control group (Figure 4D).

Administration of chloroquine via injection resulted in a marked enhancement of the regenerative effects of transplanted CDSCs on tendon tissue. Notably, approximately half of the samples displayed no signs of heterotopic ossification, while the remaining samples exhibited only mild ossification (Figure 4E). However, co-administration of AZD4547, an FGF inhibitor, with chloroquine (CQ + AZD group) led to a significant reappearance of heterotopic ossification, suggesting that FGF signaling inhibition abrogated the beneficial effects of chloroquine (Figure 4F). Histological analysis of stained sections revealed significant differences between the chloroquine group (CQ) and the other 3 groups. The CQ group displayed the lowest scores for fiber structure, fiber arrangement, nuclear roundness, and the presence of heterotopic ossification, as assessed using a semi-quantitative grading scale. Statistical analysis confirmed these observations, demonstrating significant differences in all parameters except cell density between the CQ group and the other groups (Figure 4G).

**Figure 5.**
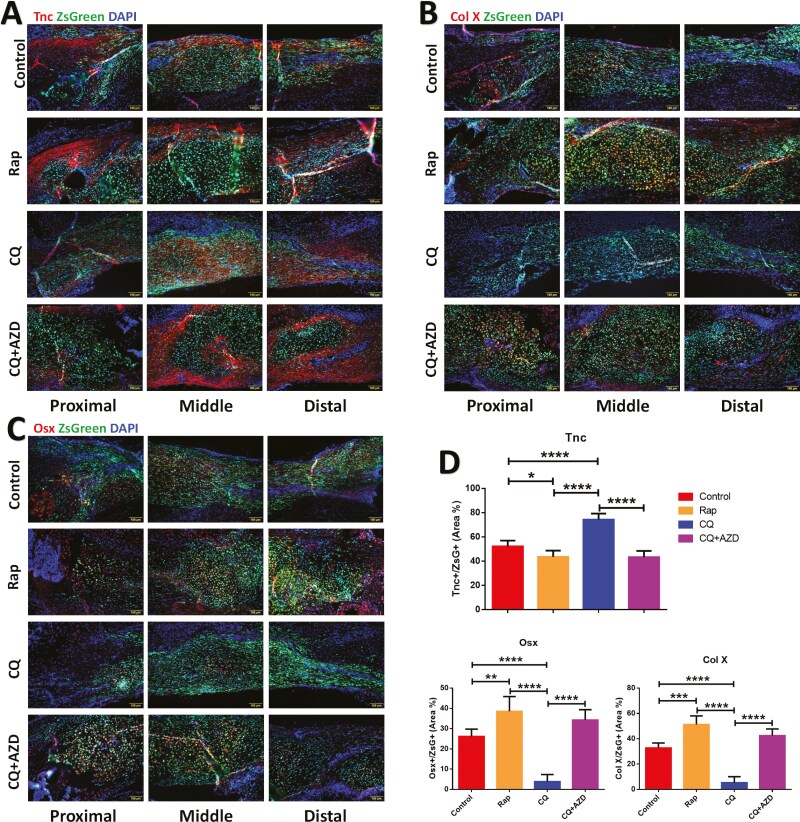
Representative immunofluorescent (IF) stains, and fluorescence quantification of each group that transplanted untreated CDSC. (A-C) 6-weeks post-transplantation, IF stains of Tnc (A) to evaluate tenogenesis, and Col X (B), Osx (C) to evaluate heterotopic ossification. Scale bar = 100 μm. (D) Fluorescent quantification of positive stained Tnc, Osx, and Col X in the ZsGreen+ area of each group (*n* = 5). Data are mean ± S.D. *, *P* < .05; **, *P* < .01; ***, *P* < .001; ****, *P* < .0001. Images of A-C are representative of 5 independent experiments.

To further assess the efficacy of tendon regeneration and the extent of heterotopic ossification following CDSC transplantation in each group, we employed immunofluorescence staining for Tnc, ColX, and Osx, which are specific markers for tendon, hypertrophic cartilage, and osteoblasts, respectively. In the control group, approximately 50% of the ZsGreen + area exhibited positive staining for Tnc (Tnc+/ZsGreen+), indicating the presence of tendon tissue. However, around 30% and 25% of the area stained positive for Col X and Osx, respectively, suggesting the presence of hypertrophic cartilage and osteoblast activity (Figure 5A-D). Intraperitoneal administration of rapamycin to induce autophagy activation enhanced chondrogenesis while attenuating tenogenesis in the transplanted CDSCs. The proportion of Tnc+/ZsGreen+ cells decreased to approximately 40% in the Rap group, whereas Col X+/ZsGreen+ and Osx+/ZsGreen+ populations increased to around 50% and 40%, respectively, signifying a shift towards a hypertrophic cartilage and bone phenotype (Figure 5A-D). Conversely, chloroquine administration via intraperitoneal injection to inhibit autophagy resulted in augmented tenogenesis and diminished chondrogenesis. The percentage of Tnc+/ZsGreen+ cells in the CQ group increased to over 75%, indicating extensive tendon formation, while Col X+/ZsGreen+ and Osx+/ZsGreen+ decreased to less than 10% (Figure 5A-D). Importantly, the pro-tenogenic effects of autophagy inhibition were abrogated by AZD4547 (CQ+ AZD group). This co-administration restored the positive area percentages for these markers to levels similar to those observed in the Rap group, suggesting a reversal of the chloroquine-induced shift toward tenogenesis.

Our in vivo findings provide compelling evidence for the modulatory effects of autophagy activation and inhibition on the tenogenic differentiation of CDSCs within a tension microenvironment. Furthermore, these results highlight the critical role of FGF signaling during the tenogenic differentiation process of CDSCs.

### Effects of post-transplant autophagy or FGF signaling interventions on tendon regeneration following transplantation of pretreated CDSCs

While Figures 4 and 5 demonstrate that post-transplantation autophagy inhibition in the CQ group reduced the severity of heterotopic ossification, it did not completely prevent its occurrence. To investigate if inducing tenogenic differentiation prior to transplantation could further improve CDSC-mediated tendon regeneration, we employed a different approach. Mice received pretreated CDSCs transplanted at the patellar tendon resection site, followed by post-transplantation interventions targeting autophagy or FGF signaling. The pretreated CDSC groups included: Ten (pretreated with cyclic tension for 72 hours but no further interventions), Ten + Rap (pretreated with cyclic tension and rapamycin for 72 hours followed by post-transplantation rapamycin injection), Ten + CQ (pretreated with cyclic tension and chloroquine for 72 hours followed by post-transplantation chloroquine injection), and Ten + CQ + AZD (pretreated with cyclic tension, chloroquine, and AZD4547 for 72 hours followed by co-administration of chloroquine and AZD4547 post-transplantation).

**Figure 6.**
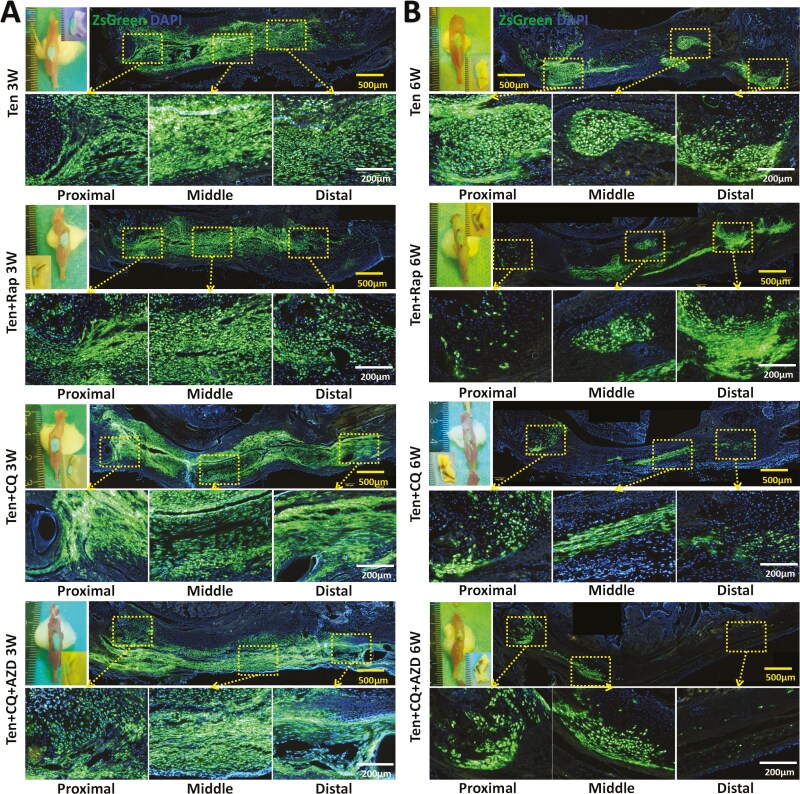
Representative gross and microscopic fluorescent images of each group that transplanted pretreated CDSC. (A, B) Gross and merged microscopic fluorescent images of each group that transplanted pretreated CDSCs after 3 weeks (A), and 6 weeks (B). Ten group, transplant cyclic tensile stress pretreated CDSCs, without post-transplant interventions; Ten + Rap group, transplant cyclic tensile stress and rapamycin pretreated CDSCs, with post-transplant rapamycin injection; Ten + CQ group, transplant cyclic tensile stress and chloroquine pretreated CDSCs, with post-transplant chloroquine injection; Ten + CQ + AZD group, transplant cyclic tensile stress, chloroquine, and AZD4547 pretreated CDSCs, with post-transplant chloroquine and AZD4547 injection. Images of A-B are representative of 5 independent experiments.

Evaluation of gross and microscopic fluorescent images revealed robust survival of the pretreated CDSCs in all groups at 3 weeks post-transplantation. Consistent green fluorescence was observed throughout the region extending from the patella to the tibial tubercle in each group, despite variations in the morphology of the transplanted cells (Figure 6A). However, due to the additional in vitro treatments the pretreated CDSCs underwent, their long-term survival at 6 weeks post-transplantation was inferior compared to the untreated group (Figure 6B). This effect was particularly evident in the Ten + CQ and Ten + CQ + AZD groups.

**Figure 7.**
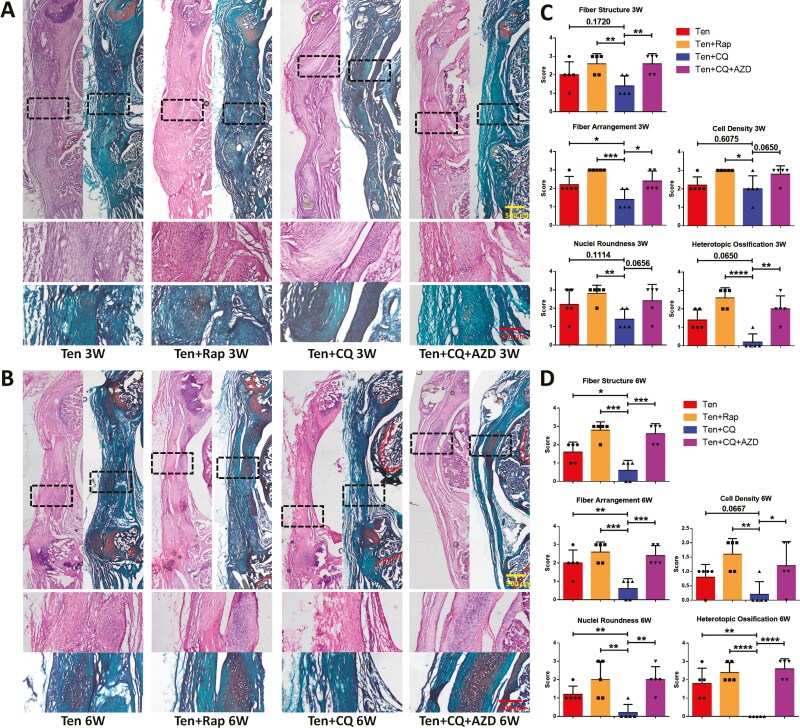
Representative H&E and Safranin O stains, and histological analyses of each group that transplanted pretreated CDSC. (A, B) Microscopic images of H&E and Safranin O stains of each group that transplanted pretreated CDSCs after 3 weeks (A), and 6 weeks (B). (C, D) 3 weeks (C), and 6 weeks post-transplantation (D), histological scores of 5 parameters to evaluate tendon regeneration of stained sections (*n* = 5) in each group. Data are mean ± S.D. *, *P* < .05; **, *P* < .01; ***, *P* < .001; ****, *P* < 0.0001. Images of A-B are representative of 5 independent experiments.

We compared tendon regeneration outcomes among different groups using histological stains and analyses. The results confirmed that the Ten + CQ group exhibited superior performance compared to the other 3 groups at both time points (Figure 7A and B). In the Ten + Rap group, activation of autophagy by rapamycin exacerbated heterotopic ossification. While the Ten + CQ group had fewer surviving ZsGreen+ cells compared to the Ten and Ten + Rap groups, there was no significant formation of heterotopic ossification detectable at the tendon resection site. Furthermore, in the Ten + CQ + AZD group, AZD4547 reactivate autophagy, thereby neutralizing chloroquine’s pro-tenogenesis effects (Figure 7A and B). Histological scores also demonstrated that CQ treatment yielded superior tendon regeneration outcomes compared to the other 3 groups at 3 weeks and 6 weeks post-transplantation (Figure 7C and D).

These findings demonstrate that pre-tenogenic induction of CDSCs prior to transplantation enhances tendon regeneration outcomes. Notably, although the Ten + CQ group exhibited superior outcomes despite harboring a limited number of surviving pre-tenogenic CDSCs at 6 weeks post-transplantation (Figures 6B and 7B), this suggests that these cells might exert additional effects beyond simple cell replacement. In addition to direct repair, one of the most significant effects of stem cell transplantation is the regulation of the local environment through paracrine mechanisms.^31-34^ Although our transplanted pre-tenogenic CDSCs did not exhibit long-term survival in the local area, they demonstrated higher expression levels of tenogenic differentiation markers and FGF pathway-related markers in our previous in vitro PCR and WB tests. We propose that the pre-tenogenic CDSCs may modulate the local microenvironment or employ paracrine signaling to promote tenogenic differentiation of endogenous migrating stem cells, thereby contributing to the observed improvement in tendon regeneration.

Our study collectively provides evidence supporting the promotion of tendon regeneration through modulation of autophagy before and after CDSC transplantation. In vitro findings revealed that tensile stress stimulation simultaneously increased the expression of tenogenic markers and activated autophagy in CDSCs. However, when CDSCs adapted to tensile stress, autophagy mediated the degradation of tenogenic proteins while preserving their chondrogenic potential. This effect was further amplified by rapamycin-induced autophagy activation. Conversely, inhibition of autophagy impeded the degradation of tenogenic proteins and concurrently activated FGF signaling, promoting tenogenic differentiation in tension-treated CDSCs (Graphic Abstract). These in vitro mechanisms were subsequently validated through a series of in vivo experiments.

## Discussion

This study demonstrated that cyclic tensile stress applied to in vitro cultured CDSCs could sustain autophagy activation. We further revealed that inhibiting autophagy with chloroquine promoted tenogenic differentiation of these tension-treated CDSCs. This effect was mediated through the activation of the Fgf2/Fgfr2 signaling pathway. Importantly, inhibition of FGF signaling by AZD4547 abrogated the pro-tenogenic effects of chloroquine and re-activated autophagy in tension-treated CDSCs. These in vitro mechanisms were subsequently validated through 2 distinct in vivo models. Our findings demonstrated that autophagy inhibition could enhance the tendon regeneration efficacy of transplanted CDSCs at the patellar tendon resection site.

Tendon injuries often result in scar formation rather than true regeneration, presenting a significant hurdle in clinical practice.^2,5^ While various stem cell types have been explored for tendon repair via transplantation, robust evidence supporting their long-term survival or direct differentiation into functional tendon tissue post-transplantation remains elusive.^5,35^ Our previous work demonstrated the potential of CDSCs for in situ biological regeneration of bone-to-tendon structures in a rat model, highlighting their osteogenic, chondrogenic, and tenogenic capacities.^24^ However, despite these promising findings, tendon regeneration outcomes remain suboptimal. Derived from cartilage, CDSCs exhibited a stronger propensity for chondrogenesis compared to tenogenesis, leading to unwanted heterotopic ossification formation in many rat models. Although tendon, bone, and cartilage share a common embryonic origin, their mature biomechanical environments differ substantially. Tendons primarily experience tensile stress as the major biomechanical force. We hypothesized that stimulating CDSCs with cyclic tension could promote tenogenic differentiation. However, our findings revealed that CDSCs could rapidly adapt to tensile stress by activating autophagy, a cellular process that maintains homeostasis and unexpectedly inhibits tenogenic differentiation. We propose that persistent autophagy activation plays a critical role in preventing CDSCs from transitioning toward a tenogenic fate under cyclic tensile stimulation.

Autophagy is a crucial regulatory mechanism by which cells maintain self-homeostasis in response to external stimuli.^36^ In stem cells, autophagy activation plays a pivotal role in maintaining stemness by inhibiting differentiation and senescence.^25,27^ Studies have shown that autophagy activation contributes to the preservation of chondrocyte-like characteristics in articular cartilage, acting as a protective mechanism against collagen degradation and osteoarthritis.^37,38^ During bone development, autophagy regulates the synthesis and secretion of type II collagen within the growth plate.^39^ However, sustained autophagy activation has deleterious effects on tendon health, altering the ultrastructure of collagen fibers and reducing mechanical strength.^40,41^ Our findings demonstrate that while rapamycin-induced autophagy in CDSCs prevents morphological changes under tensile stress, chloroquine-mediated autophagy inhibition promotes the formation of spindle-shaped cells resembling tendon stem cells. This shift from chondrogenic features towards a tenogenic phenotype is further supported by our PCR and Western blot results.

Several signaling pathways are known to regulate tendon repair and development.^42^ To elucidate the specific signaling cascade involved in the pro-tenogenic differentiation of CDSCs induced by autophagy inhibition and tensile stress, we conducted a PCR analysis. Following the exclusion of genes with undetectable expression or statistically insignificant changes, we observed activation of the FGF signaling pathway during this pro-tenogenic process. Notably, mRNA expression levels of Fgf2 and Fgfr2 exhibited a significant upregulation, highlighting their established roles as growth factors and receptors critical for tendon development.^43-45^ We subsequently employed the FGF signaling inhibitor AZD4547 and observed that it abrogated the pro-tenogenic effects of chloroquine-induced autophagy inhibition on tension-treated CDSCs. Furthermore, our data revealed that AZD4547 treatment in tension-treated CDSCs led to the reactivation of autophagy, which had been previously suppressed by chloroquine, resulting in a shift back toward a chondrogenic phenotype. These findings suggest that FGF signaling likely influences various biological processes through its modulation of autophagy. In investigations involving diverse cell types, the impact of FGF signaling pathway on autophagy exhibits variability.^39,46-48^ We postulated that the upregulation of Fgf2 and Fgfr2 contributes to the inhibition of autophagy in CDSCs, while AZD4547-mediated suppression of the FGF signaling pathway could partially restore autophagic activity. This activation effect on autophagy by AZD4547 has also been documented by other researchers.^48,49^

This study significantly contributes to the field by establishing 2 distinct CDSC transplantation paradigms and demonstrating that both pre-treated and untreated CDSCs possess the capacity to regenerate resected patellar tendons. The use of wild-type animals throughout the investigation underscores the excellent immunocompatibility of CDSCs. Furthermore, these cells exhibit robust in vivo survival and direct tendon regeneration capabilities following transplantation. Their abundant sources, ease of in vitro expansion, and outstanding immunocompatibility collectively position CDSCs as a promising therapeutic candidate for tendon regeneration. While in vitro pre-induction may lessen post-transplantation survival, pre-tenogenic induction of CDSCs further enhanced both direct and indirect regeneration of the resected tendon. Future investigations will focus on translating these cell manipulation and transplantation protocols to large animal models. Additionally, we aim to explore the tenogenic differentiation potential and in vivo tendon regeneration capabilities of human CDSCs.

Overall, our study corroborated that regulating autophagy of CDSCs in response to tensile stress could enhance post-transplant tendon regeneration. Both in vitro and in vivo results substantiated that inhibiting autophagy promotes tenogenic differentiation of CDSCs when stimulated by tensile stress, which was mediated through the activation of FGF signaling. Inducing autophagy or inhibiting FGF signaling impeded the tenogenic differentiation of CDSCs, resulting in excessive formation of heterotopic ossification after transplantation. Indeed, our findings hold significant value for advancing the development of biological regeneration techniques for tendon injuries. However, further rigorous experiments using large animal models and preclinical studies are still required to validate their efficacy and safety in a clinical setting.

## Supplementary material

Supplementary material is available at *Stem Cells Translational Medicine* online.

szae085_suppl_Supplementary_Figure_S1-5_S7

## Data Availability

The datasets generated during and/or analyzed during the current study are available from the corresponding author upon reasonable request.
